# hLMR1, a hepatocyte-specific long noncoding RNA that represses amino acid catabolism through pre-mRNA interaction in human liver

**DOI:** 10.1371/journal.pone.0353674

**Published:** 2026-07-21

**Authors:** Marcos E. Jaso-Vera, Shohei Takaoka, Lanuza A.P. Faccioli, Zhiping Hu, Alejandro Soto-Gutierrez, Xiangbo Ruan

**Affiliations:** 1 Division of Endocrinology, Diabetes and Metabolism, Johns Hopkins University School of Medicine, Baltimore, Maryland, United States of America; 2 Institute for Fundamental Biomedical Research, Johns Hopkins All Children’s Hospital, St. Petersburg, Florida, United States of America; 3 Department of Pathology, University of Pittsburgh, Pittsburgh, Pennsylvania, United States of America; 4 Center for Transcriptional Medicine, University of Pittsburgh, Pittsburgh, Pennsylvania, United States of America; Anhui University of Chinese Medicine, CHINA

## Abstract

Amino acid (AA) catabolism and ureagenesis in the liver are essential for maintaining systemic nitrogen homeostasis. Patients with Metabolic Dysfunction-Associated Steatotic Liver Disease (MASLD) exhibit impaired hepatic AA catabolism and urea production, accompanied by repression of genes governing these pathways. The molecular basis of this repression remains unknown. Here, we identify the hepatocyte-specific long noncoding RNA hLMR1 as a key suppressor of hepatic AA catabolism. Using a humanized liver mouse model, we show that hLMR1 knockdown broadly upregulates genes involved in AA degradation and ureagenesis. Chromatin isolation by RNA purification (ChIRP) coupled with RNA-seq reveals extensive interactions between hLMR1 and the pre-mRNAs of AA catabolism genes, mediated by a complementary motif within hLMR1 (nucleotides 587–598). In primary human hepatocytes, hLMR1 overexpression inhibits glucagon-induced activation of AA catabolic genes, whereas deletion of the 587–598 region abrogates this effect. Analysis of human liver RNA-seq datasets demonstrates a negative correlation between hLMR1 expression and AA catabolic gene programs in MASLD. Together, these findings uncover hLMR1 as a previously unrecognized regulator of hepatic nitrogen metabolism, linking lncRNA dysregulation to metabolic dysfunction in MASLD.

## Introduction

The liver plays a central role in maintaining whole-body nitrogen homeostasis. It is the primary site for catabolizing non-branched amino acids, including alanine, serine, glycine, glutamine, and histidine. Amino acid catabolism is tightly coupled to ureagenesis, a process that converts ammonia derived from amino acid deamination into urea for renal excretion [1]. In chronic metabolic diseases such as Metabolic Dysfunction-Associated Steatotic Liver Disease (MASLD) and type 2 diabetes (T2D), hepatic capacity for amino acid catabolism and urea synthesis is markedly impaired [2–5]. Indeed, expression of key enzymes involved in nitrogen metabolism, including SDS, GLS2, CPS1, and ASS1, is significantly reduced in the livers of MASLD patients [2,6]. As a result, MASLD patients often exhibit elevated circulating amino acid levels and hyperglucagonemia, likely driven by amino acid–stimulated glucagon release from pancreatic α-cells [7,8]. This dysregulated liver–α-cell axis has emerged as a potential contributor to the pathogenesis of MASLD and T2D [3,4,9]. However, the molecular mechanisms underlying the suppression of hepatic amino acid catabolism in humans remain poorly understood.

Long non-coding RNAs (lncRNAs) constitute a large class of RNA transcripts that exert regulatory control over diverse metabolic pathways [10–19]. Although lncRNA functions have been demonstrated in animal models, most human lncRNAs are poorly conserved, limiting mechanistic studies in mice [20]. To overcome this challenge, we employed a humanized liver mouse model [21] in which human hepatocytes replace most mouse hepatocytes, allowing investigation of human-specific lncRNAs in vivo [14,15]. Using this system, we previously identified a human-specific lncRNA, hLMR1 (human lncRNA metabolic regulator 1), which promotes hepatic cholesterol biosynthesis [15]. In the present study, RNA-seq analysis of humanized livers following hLMR1 knockdown revealed that hLMR1 represses a broad set of genes involved in amino acid catabolism and ureagenesis. Mechanistically, hLMR1 extensively interacts with the pre-mRNAs of amino acid catabolic genes, and this lncRNA–pre-mRNA interaction is essential for its repressive function. Consistent with this finding, analysis of human liver transcriptomes from MASLD patients showed that hLMR1 expression negatively correlates with amino acid catabolism gene expression. Together, our results identify hLMR1 as a key suppressor of hepatic amino acid catabolism and suggest that its upregulation may contribute to nitrogen metabolic dysfunction in MASLD.

## Methods

### Bulk RNA-seq analysis of humanized liver mouse data

Bulk RNA-seq samples were processed by the Illumina stranded total RNA ligation prep kit and sequenced on the Illumina NovaSeq S1 200 platform at The Single Cell & Transcriptomics Core of Johns Hopkins School of Medicine. The quality of raw sequencing reads (FASTQ files) was assessed using FastQC (version 0.11.8). Adapter sequences and low-quality reads were trimmed or removed using Trimmomatic (version 0.39) [22]. Prior to alignment, the GRCh38 Ensembl human genome and the GRCm39 Ensembl mouse genome were merged to enable host–graft read discrimination. Filtered reads were aligned to the merged genome using STAR (version 2.7.8a) [23]. Uniquely mapped human reads were extracted from the resulting BAM files using SAMtools (version 1.13) [24]. Gene-level read counts for mature mRNA were generated with featureCounts from the Subread package (version 2.0.0) [25]. Genes expressed at raw counts greater than one in two or fewer samples were excluded prior to normalization. For data quality assessment, the count matrix was analyzed in R (version 4.1.0) using DESeq2 (version 1.36.0) [26]. Variance stabilizing transformation (VST) was applied, and principal component analysis (PCA) was performed using the DESeq2 workflow.

The count matrix was normalized and analyzed for differential expression in R using DESeq2. Due to high biological variability among humanized liver samples, differentially expressed genes (DEGs) were defined as those with P ≤ 0.05 and an absolute log2 fold change (|Log2FC|) ≥ 0.38. For GSEA, genes were first filtered to retain the top 10,000 genes by baseMean across all genes, including DEGs. The filtered gene list was then ranked by log2 fold change and analyzed using clusterProfiler (version 4.12.6) [27] with default settings. Raw sequencing data supporting the findings of this section have been deposited in the NCBI Gene Expression Omnibus (GEO) database under accession number GSE335366.

### Chromatin isolation by RNA purification (ChIRP)

The ChIRP protocol in this study is based on the previous publication [28] with slight modifications. Pooled human liver tissues from eight donors (4 male and 4 female) were minced into thin slices and crosslinked in 3% formaldehyde (1 mL per 50 mg tissue) (Thermo Cat#28906) for 30 minutes at room temperature. Crosslinking was quenched by adding 0.125 mM glycine (CST Cat#7005S) and incubating for 5 minutes. The tissue was pelleted at 2,000 × g, washed once with PBS, pelleted again, and the supernatant carefully aspirated. The pellet was resuspended in cell lysis buffer (100 mg tissue per 1 mL buffer, 50 mM Tris-HCl pH 7.0, 10 mM EDTA, and 1% SDS). Cells were disrupted using a tissue grinder to release crosslinked RNA, DNA, and protein complexes.

Chromatin was sheared by sonication using a Bioruptor (Diagenode Cat#B01020001) for 1 hour. To confirm appropriate shearing, a 5 µL aliquot was purified using a PCR purification kit and analyzed by agarose gel electrophoresis (Qiagen Cat#28106), ensuring an average fragment size below 500 bp. The lysate was clarified by centrifugation at 16,000 × g for 10 minutes at 4 °C, and the supernatant was transferred to fresh tubes.

For hybridization, samples were diluted threefold in hybridization buffer (750 mM NaCl, 1% SDS, 50 mM Tris-HCl pH 7.0, 1 mM EDTA, and 15% formamide) and incubated with biotinylated RNA-specific ChIRP probes (1 µL of 100 µM probe mix per 1 mL lysate). Two independent probe libraries targeting unique regions of the hLMR1 transcript were used (**S3 Table**). A probe library targeting LINC01018 served as a negative control. Hybridization was carried out at 37 °C for 16 hours with end-to-end rotation.

After hybridization, 100 µL of C-1 Dynabeads (Invitrogen Cat#65001) were added per 1 µL of 100 µM probes and incubated at 37 °C for 30 minutes. Beads were then washed five times with wash buffer and transferred to a new tube for RNA extraction. RNase A-treated samples were included as negative controls, and input lysates served as positive controls.

To recover RNA, bead-bound and input samples were treated with proteinase K (5 µL in 95 µL RNA PK buffer, 100 mM NaCl, 10 mM Tris-HCl pH 8.0, 1 mM EDTA, and 0.5% SDS) for 45 minutes at 50 °C with end-to-end shaking, followed by heat inactivation at 95 °C for 10 minutes. RNA was then extracted using Trizol–chloroform, and the aqueous phase was purified using RNeasy Mini columns following the manufacturer’s clean-up and on-column DNase digestion protocols (Qiagen Cat#74106). Purified RNA was eluted in nuclease-free water and used for cDNA synthesis. ChIRP efficiency was evaluated by qPCR using oligonucleotides specific to hLMR1 and LINC01018 (negative control).

### Chromatin isolation by RNA purification (CHIRP) followed by RNA-seq analysis

ChIRP-RNA samples were processed by the Takara SMARTer stranded v3 total RNA library prep kit and sequenced on the Illumina NovaSeq S1 200 platform at The Single Cell & Transcriptomics Core of Johns Hopkins School of Medicine. Sequencing read quality was assessed using FastQC (version 0.11.8), and adapter sequences and low-quality reads were removed with fastp (version 0.23.2) [29]. Filtered reads were aligned to the GRCh38 Ensembl human genome using STAR (version 2.7.8a). Reads with mapping quality scores below 30 were excluded using SAMtools (version 1.13). PCR duplicates were removed with Picard (version 2.27.4) [RRID: SCR_006525].

ChIRP-enriched RNA peaks were identified using MACS2 (version 2.2.7.1) [30] in single-end mode with matched input controls. To optimize peak detection, the mfold lower and upper parameters were adjusted to 2 and 100, respectively (default values: 5 and 50). Peaks overlapping ENCODE Blacklist regions for hg38 were excluded using bedtools (version 2.24.0) [31]. Remaining peaks were annotated using the annotatePeaks.pl command from HOMER (version 4.11) [32].

Only peaks overlapping between two independent probe sets targeting hLMR1, while not overlapping with peaks derived from LINC01018-targeting oligos, were retained using bedtools filtering. The resulting high-confidence hLMR1-interacting RNA peaks were analyzed for regulatory motif enrichment with the findMotifsGenome.pl command from HOMER. The top 1000 filtered peaks, ranked by MACS2 peak score, were further used for KEGG pathway enrichment analysis using DAVID [33]. Raw sequencing data supporting the findings of this section have been deposited in the NCBI Gene Expression Omnibus (GEO) database under accession number GSE335367.

### Adenovirus construction, production, and titration

Full-length hLMR1 and deletion mutant hLMR1Δ587–598 sequences were cloned into the pAd/CMV/V5-DEST vector using the Gateway cloning system (Invitrogen). PCR amplification was used to introduce attB recombination sites into the hLMR1 sequence derived from pCDNA3.1 plasmids (**S3 Table**). The amplified DNA fragments were purified from a 1% agarose gel and cloned into the pDONR221 entry vector using the BP recombination reaction according to the manufacturer’s instructions (Invitrogen Cat#12536017 and Cat#11789021). LR recombination was then performed to transfer the inserts into the pAd/CMV/V5-DEST destination vector (Invitrogen Cat#V49320). The integrity of all plasmids was verified by whole-plasmid sequencing.

For adenovirus production, six 15-cm dishes of 293A cells were transfected with 2 µg of PacI-linearized pAd/CMV/V5-DEST–hLMR1 or Δ587–598 plasmids using Lipofectamine 2000 (Thermo Fisher Scientific, Cat#11668019), following the manufacturer’s protocol. Two days post-transfection, cells were transferred to 10-cm dishes, and the medium was replaced every two days until a clear cytopathic effect was observed under the microscope. The cells and medium were collected when more than 80% of the cells had detached, and three freeze–thaw cycles were performed to release the crude virus.

For virus amplification and purification, 293A cells were infected with the crude viral lysate and incubated for 48 hours. Cells were harvested, resuspended in PBS, and subjected to three additional freeze–thaw cycles to release viral particles. The lysate was centrifuged to remove cell debris, and the supernatant was purified using a 50% cesium chloride (CsCl) gradient (32,000 × g, 4 °C, 18 h). The viral band was collected from the top of the gradient using an 18-gauge syringe, it was desalted by gravity flow through a PD-10 desalting column (GE Healthcare) and quantified by measuring OD260/280 with a Cytation spectrophotometer. The virus was diluted to a final concentration of 1 × 10^12^ particles/mL and stored at −80 °C until use.

Virus titration was performed using the Adeno-X Rapid Titer Kit (Takara Bio, Cat#632250). Briefly, 293A cells were seeded in 24-well plates and infected with serial dilutions (10 ^−^ ^2^, 10 ⁻ ^4^, 10 ^−^ ^5^, 10 ^−^ ^6^, and 10 ^−^ ^7^) of the purified virus. After 48 hours of incubation, cells were fixed with ice-cold methanol and washed three times with PBS containing 1% BSA. An anti-hexon antibody was applied and incubated for 1 hour at 37 °C, followed by three PBS washes. A horseradish peroxidase (HRP)-conjugated rat anti-mouse secondary antibody was then applied for 1 hour at 37 °C. After washing three times with PBS containing 1% BSA, a DAB working solution was added to visualize infection foci. Black-stained foci were counted at 20 × magnification, and infectious units (IFU/mL) were calculated for each dilution. The average IFU/mL across dilutions was used as the final virus titer, which was applied in subsequent experiments.

### Primary human hepatocyte (PHH) culture

Diseased adult human liver cells were obtained from the Human Organ processing hub and from the Center for Transcriptional Medicine at the University of Pittsburgh. The Institutional Review Board at the University of Pittsburgh has approved our protocol and given the Not Human Research Determination (IRB# STUDY24020093). Hepatocytes were isolated using a three-step collagenase digestion technique as previously described [34]. Cells were kept on ice throughout handling until plating. Cell suspensions were gently resuspended and diluted fourfold with ice-cold Williams’ E Medium (WEM) (Thermo Fisher Scientific, Cat#A1217601). The diluted cells were centrifuged at 600 rpm for 7 minutes at 4 °C, and the pellet was resuspended in ice-cold WEM followed by a second centrifugation at 500 rpm for 5 minutes at 4 °C. After washing, the cell pellet was resuspended in 10 mL of ice-cold WEM supplemented with 5% FBS. Viable cells were counted using a Neubauer hemocytometer with trypan blue exclusion. Cells were then diluted in WEM containing 10% FBS at 37 °C to a final concentration of 1 × 10⁶ cells/mL. A total of 0.5 mL of the suspension was plated per well in a 24-well plate pre-coated with collagen. After 3 hours of attachment, cells were washed twice with PBS, and pre-warmed WEM containing the designated treatments was added. Cultures were maintained at 37 °C in 5% CO_2_.

### Primary mouse hepatocyte (PMH) culture

Primary mouse hepatocytes were isolated from C57BL/6J mice by in situ perfusion. The liver was perfused via the vena cava with 30 mL of pre-warmed perfusion buffer (HBSS containing 0.5 mM EDTA, pH 8.0, and 25 mM HEPES, pH 7.5) at a flow rate of 1 mL/min. Immediately afterward, 30 mL of pre-warmed digestion buffer (HBSS containing 25 mM HEPES, pH 7.5, and 1 mg/mL Collagenase D) was perfused at the same rate. The digested liver was excised and placed in ice-cold DMEM supplemented with 10% FBS. Hepatocytes were gently released by teasing the liver capsule with forceps, and the resulting cell suspension was filtered through a 100 µm cell strainer. Cells were pelleted by centrifugation at 50 × g for 5 minutes at 4 °C, resuspended in fresh DMEM + 10% FBS, and washed twice by repeating the centrifugation step. The final pellet was resuspended in 5 mL of DMEM + 10% FBS and kept on ice. Cell number and viability were determined using trypan blue exclusion. Cells were diluted in WEM + 10% FBS at 37 °C to a final concentration of 1 × 10⁶ cells/mL, and 0.5 mL of the suspension was added to each well of a collagen-coated 24-well plate. After 3 hours of attachment, cells were washed twice with PBS and incubated in pre-warmed WEM containing the required treatments. Cultures were maintained at 37 °C in 5% CO_2_.

### Primary hepatocyte transduction and treatment

For viral transduction, adenoviral vectors were added to WEM containing 10% FBS at a multiplicity of infection (MOI) of 20. The virus-containing medium was added to the hepatocyte cultures and incubated for 24 hours. Cells were then washed twice with PBS and replenished with fresh sterile WEM + 10% FBS, followed by overnight incubation under the same conditions. The next morning, PHH were washed twice with PBS and incubated with WEM containing 2.5 mM glucose for 3 hours. Cells were then washed twice with PBS and treated with either WEM containing 2.5 mM glucose or WEM supplemented with 50 nM glucagon and 2 mM pyruvate, as described in the text. PHH were incubated for 11 hours, after which the culture medium was collected and stored at −80 °C. Cells were washed once with ice-cold PBS and lysed in RLT buffer for RNA extraction using the RNeasy Mini Kit.

### Complementary DNA synthesis and quantitative PCR (RT-qPCR)

Total RNA was extracted using the RNeasy Mini Kit with on-column DNase digestion. RNA was eluted in nuclease-free water, and 500 ng of RNA per sample was used for cDNA synthesis with SuperScript III Reverse Transcriptase (Thermo Fisher, Cat#11752050). The resulting cDNA was diluted 1:10 in nuclease-free water. Quantitative PCR was performed using SYBR Green reagents on a QuantStudio 7 instrument. Primer sequences including the human-specific primers for qPCR are listed in (**S3 Table**).

### Urea quantitation in cell culture media

Culture media from PHH experiments were thawed on ice and directly analyzed for urea content using the Urea Assay Kit (Abcam, Cat# ab83362), following the manufacturer’s instructions. Absorbance was measured using a Cytation microplate reader, and urea concentrations were calculated according to the standard curve.

### Animal experiments

Humanized liver tissues with or without hLMR1 knocking down were obtained from the PI’s previous study following the experimental procedure as described [15]. Isolation of primary mouse hepatocytes was approved by the Johns Hopkins University Animal Care and Use Committee (ACUC) with the approval number MO24C313. During the isolation, mice were anesthetized using ISOFLURANE inhalant anesthesia (3–4% for induction and 1–3% for maintenance). Mice were euthanized by exsanguination during the perfusion of isolation buffers.

### Statistics

Statistical analysis was performed using R version 4.5.0. Student’s t-test was used for comparisons between two groups; One-way ANOVA was used for comparisons between three or more groups, followed by post hoc analysis with Tukey-HSD. ANOVA p-values reported in each bar chart represent the overall significance of the differences among the groups being compared. Significant differences among each individual group were annotated by line illustrations and stars, which were automatically generated in R.

## Results

### hLMR1 suppresses amino acid catabolism and ureagenesis in humanized liver mice

**Fig 1:**
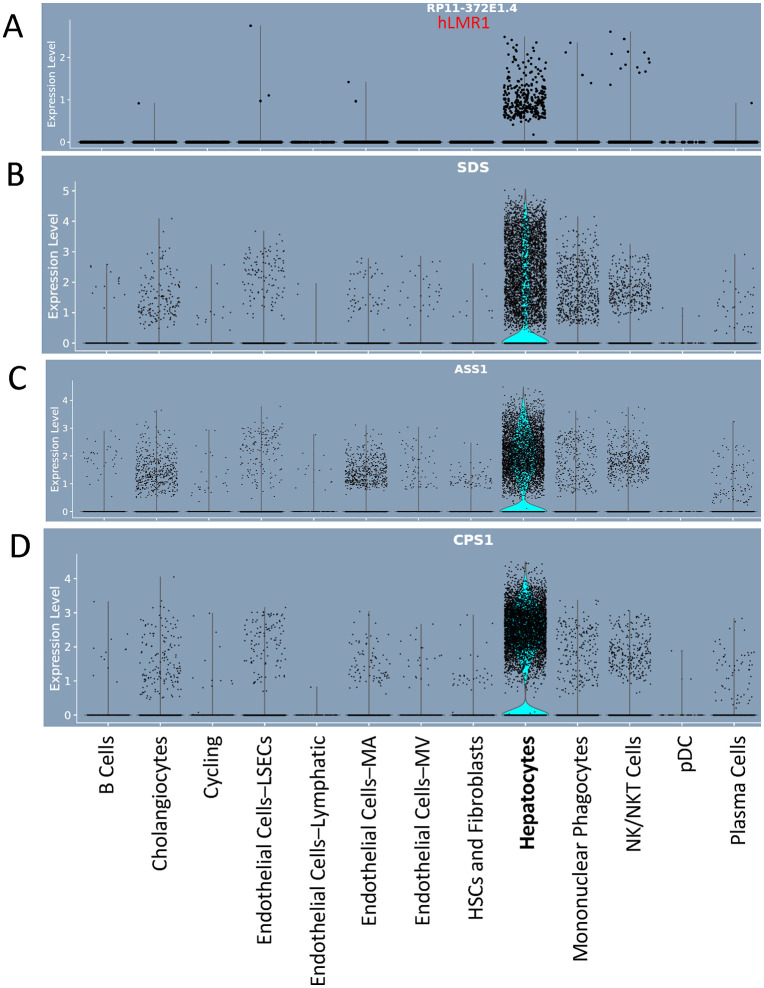
Expression of hLMR1 (A), SDS (B), ASS1 (C) and CPS1 (D) in major cell groups of human livers. Data were adapted from http://liveratlas-vilarinholab.med.yale.edu/.

We previously identified hLMR1 as a non-conserved, liver-specific human lncRNA that is induced by feeding and promotes cholesterol synthesis [15]. Single-cell RNA-seq data from human liver tissues [35] revealed that hLMR1, along with key amino acid (AA) catabolism genes, was highly enriched in hepatocytes (**Fig 1**). To systematically identify genes regulated by hLMR1, we performed high-throughput RNA sequencing on humanized mouse livers with or without hLMR1 knockdown. To specifically analyze the human transcriptome, RNA-seq reads were aligned to a combined human–mouse reference genome, and only human-specific reads were used for differential gene expression analysis (**Methods**). Principal Component Analysis (PCA) showed that PC1 explained the majority of the variance between the control sh-LacZ and sh-hLMR1 groups. Although one sh-LacZ sample displayed greater variability compared with the other control samples, overall group separation was maintained (**S1 Fig**).

**Fig 2:**
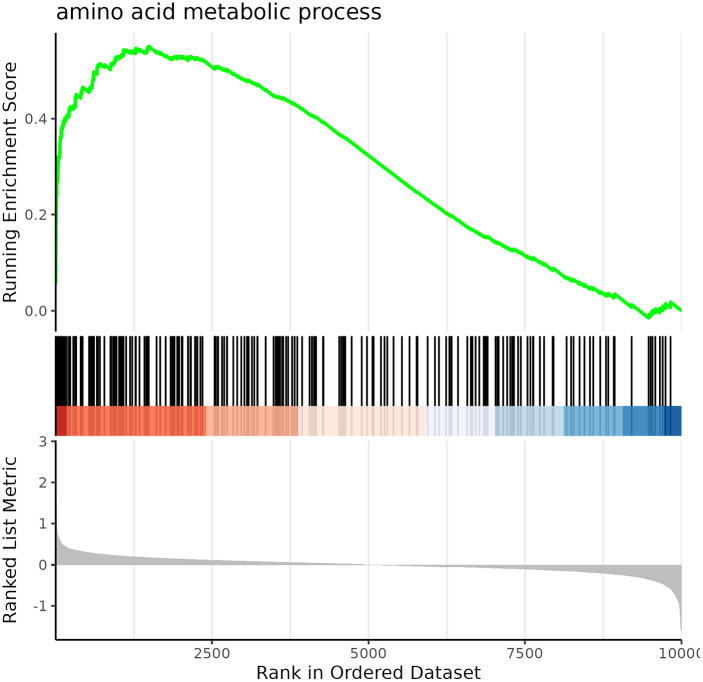
Representative Gene set enrichment analysis (GSEA) for the “amino acid metabolic process” gene set. Top: running enrichment score (green) across the ranked gene list; the early peak shows the set is enriched near the top. Middle: black ticks mark where gene set members appear in the ranked list; the color bar below shows gene score direction (red = high, blue = low). Bottom: the ranked list metric (gray) used to order genes.

As expected, hLMR1 knockdown reduced the expression of genes involved in cholesterol and fatty acid synthesis (**Supplementary Table 1**). Notably, hLMR1 depletion also led to robust upregulation of genes associated with amino acid metabolism and ureagenesis (**Table 1** and **Fig 2**). These included enzymes central to amino acid catabolism—such as serine dehydratase (SDS), glutaminase 2 (GLS2), and aminoadipate-semialdehyde synthase (AASS)—as well as urea cycle enzymes, including argininosuccinate synthase 1 (ASS1), carbamoyl-phosphate synthase 1 (CPS1), and arginase 1 (ARG1) (**Table 2**). To experimentally validate the RNA-seq findings, we designed human-specific qPCR primers and quantified the expression of hLMR1 and the top-regulated amino acid catabolism genes. We found the qPCR results were largely consistent with the RNA-seq analysis (**S2 Fig**).

**Table 1:** Top 10 GO terms from the Gene set enrichment analysis (GSEA) using the gene expression data generated from RNA-seq analysis of humanized livers receiving sh-LacZ (n = 4) and sh-hLMR1 (n = 5).

ID	Description	Enrichment Score	NES	Adjusted P-Value
GO:0006520	amino acid metabolic process	0.55	2.55	2.45E-15
GO:1901605	alpha-amino acid metabolic process	0.60	2.67	7.58E-15
GO:0170033	L-amino acid metabolic process	0.62	2.64	5.39E-13
GO:0170039	proteinogenic amino acid metabolic process	0.63	2.69	1.27E-12
GO:0009063	amino acid catabolic process	0.67	2.68	1.47E-11
GO:1901606	alpha-amino acid catabolic process	0.68	2.67	2.25E-09
GO:0170040	proteinogenic amino acid catabolic process	0.73	2.60	6.16E-08
GO:0170035	L-amino acid catabolic process	0.71	2.58	2.99E-07
GO:1901607	alpha-amino acid biosynthetic process	0.64	2.42	2.79E-06
GO:0009066	aspartate family amino acid metabolic process	0.67	2.31	1.85E-05

**Table 2:** Representative genes in the AA catabolism and ureagenesis pathway that were upregulated by knocking down of hLMR1 in humanized livers. N = 4 for sh-LacZ and n = 5 for sh-hLMR1.

Gene	Function	log2FoldChange (sh-hLMR1 vs sh-LacZ)	P-Value
SDS (serine dehydratase)	Metabolizing serine, glycine and threonine	2.801156789	0.00013531
GLS2 (glutaminase 2)	Hydrolysis of glutamine	1.430426435	0.01392321
AASS (aminoadipate-semialdehyde synthase)	Lysine degradation	1.002846532	0.00059062
HAL (histidine ammonia-lyase)	Histidine catabolism	0.873369336	0.00516139
HPD (4-hydroxyphenylpyruvate dioxygenase)	Catabolism of tyrosine	0.68933997	0.00530736
HGD (homogentisate 1,2-dioxygenase)	Catabolism of tyrosine and phenylalanine	0.772817664	0.00031782
GPT2 (glutamic--pyruvic transaminase 2)	Catabolism of alanine	0.508864917	0.03907781
ASS1 (argininosuccinate synthase 1)	Urea cycle	0.717829044	0.00199932
CPS1 (carbamoyl-phosphate synthase 1)	Urea cycle	0.572249754	0.00328639
ARG1 (arginase 1)	Urea cycle	0.416535001	0.01049737

Together, these data reveal that, beyond promoting lipid synthesis, hLMR1 functions as a repressor of amino acid catabolism and ureagenesis in human hepatocytes.

### hLMR1 interacts with pre-mRNAs of amino acid catabolism and ureagenesis genes

**Fig 3:**
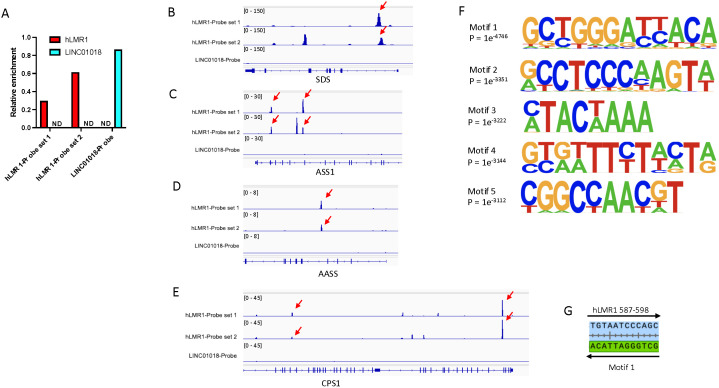
A. Relative enrichment of hLMR1 or LINC01018 in the ChIRP analysis. Relative enrichment is defined by the ratio between recovered portion and input. ND, not detected. **B-E**. Representative hLMR1 ChIRP RNA-seq peaks in SDS, ASS1, AASS, and CPS1. Peaks were visualized using CPM normalized bigwig files in IGV. Top overlapped peaks between hLMR1 probe sets were pointed by red arrows. ChIRP RNA-seq for another highly expressed lncRNA, LINC01018 was used as a negative control. The same peak-calling thresholds were used for the hLMR1 and LINC01018 datasets. hLMR1 peaks were defined as reproducible if peaks overlapped by at least 1 bp between replicates, after which overlapping peaks were merged. Peaks overlapping the LINC01018 control dataset by at least 1 bp were then excluded. All ChIRP experiments were performed using pooled human liver tissues. **F**. Top 5 motifs analyzed by HOMER (Hypergeometric Optimization of Motif EnRichment) using the sequences of hLMR1 ChIRP RNA-seq peaks. **G**. Illustration of hLMR1 sequence 587-598 matching with Motif 1.

To explore the molecular basis of hLMR1-mediated repression, we performed Chromatin Isolation by RNA Purification (ChIRP) [36] followed by RNA sequencing on human liver tissues. This approach enabled purification of endogenous hLMR1 and identification of RNA molecules interacting with it. To reduce interindividual variability, we pooled cryo-pulverized liver tissues from four male and four female donors. We used two independent biotinylated oligo sets covering most of the hLMR1 transcript, and one control oligo set targeting the abundant lncRNA LINC01018 [14]. Real-time PCR confirmed specific enrichment of hLMR1 and LINC01018 by their corresponding probes (**Fig 3A**), validating the ChIRP assay. Only ChIRP signals that overlapped between both hLMR1 oligo sets but absent in LINC01018 controls were defined as hLMR1-interacting RNA peaks (**Methods**).

Strong hLMR1 ChIRP signals were detected across the intronic regions of genes involved in amino acid catabolism and ureagenesis (Figs 3B–3E). KEGG pathway analysis of the top 1,000 hLMR1-interacting genes confirmed enrichment in amino acid metabolic pathways (**Table 3** and **S2 Table**). Motif analysis revealed several enriched RNA motifs (**Fig 3F**), among which the top motif (GCTGGGATTACA) was perfectly complementary to the hLMR1 sequence at nucleotides 587–598 (**Fig 3G**). These findings suggest that hLMR1 interacts directly with the pre-mRNAs of its target genes through a conserved complementary motif.

**Table 3:** KEGG pathway analysis using the list of top 1000 genes with peaks in hLMR1 ChIRP RNA-seq analysis.

Gene	Function	log2FoldChange (sh-hLMR1 vs sh-LacZ)	P-Value
SDS (serine dehydratase)	Metabolizing serine, glycine and threonine	2.801156789	0.00013531
GLS2 (glutaminase 2)	Hydrolysis of glutamine	1.430426435	0.01392321
AASS (aminoadipate-semialdehyde synthase)	Lysine degradation	1.002846532	0.00059062
HAL (histidine ammonia-lyase)	Histidine catabolism	0.873369336	0.00516139
HPD (4-hydroxyphenylpyruvate dioxygenase)	Catabolism of tyrosine	0.68933997	0.00530736
HGD (homogentisate 1,2-dioxygenase)	Catabolism of tyrosine and phenylalanine	0.772817664	0.00031782
GPT2 (glutamic--pyruvic transaminase 2)	Catabolism of alanine	0.508864917	0.03907781
ASS1 (argininosuccinate synthase 1)	Urea cycle	0.717829044	0.00199932
CPS1 (carbamoyl-phosphate synthase 1)	Urea cycle	0.572249754	0.00328639
ARG1 (arginase 1)	Urea cycle	0.416535001	0.01049737

### hLMR1 suppresses glucagon-induced amino acid catabolism in primary human hepatocytes

**Fig 4:**
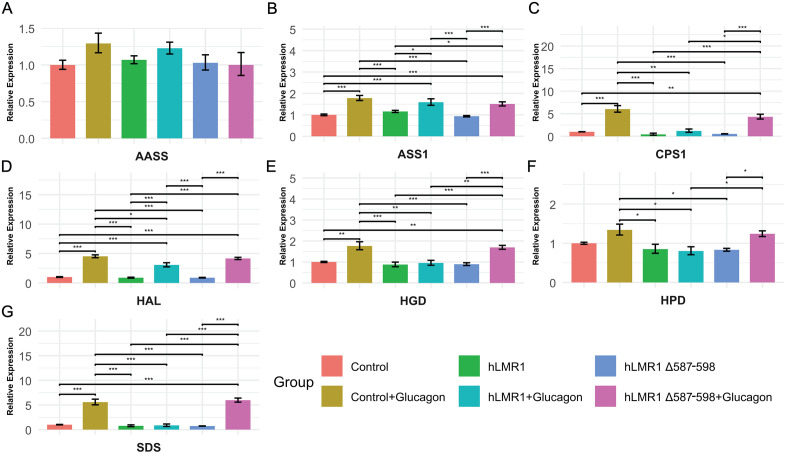
A-G. Relative mRNA expression of AASS, ASS1, CPS1, HAL, HGD, HPD and SDS in primary human hepatocytes (donor 1) infected with empty adenoviruses (Control) or adenoviruses expressing hLMR1, or hLMR1Δ587-598. 36 hours after adenovirus infection, cells were treated with or without glucagon (50nM) for 10 hours and RNA were extracted for gene expression analysis. Data are shown as the geometric mean ± SEM (n = 3-4 for each group). One-way ANOVA statistical analysis and Tukey HSD test were performed to calculate p-values. *, p < 0.05, **, p < 0.01, ***, p < 0.001.

To test the functional significance of the hLMR1–pre-mRNA interaction, we generated adenoviruses expressing either wild-type hLMR1 or a mutant lacking the 587–598 motif (hLMR1Δ587–598). Primary human hepatocytes were infected with control, hLMR1, or hLMR1Δ587–598 adenoviruses and treated with glucagon for 10 hours. In control hepatocytes, glucagon robustly induced expression of SDS, CPS1, ASS1, HGD, and HAL, consistent with its known role in stimulating amino acid catabolism (**Fig 4**). Overexpression of hLMR1 completely blocked glucagon-induced SDS, CPS1, and HGD expression (**Figs 4C**, **4E & 4G**), whereas induction of ASS1 and HAL was largely retained (**Figs 4B** & **4D**). In contrast, hLMR1Δ587–598 had no inhibitory effect, and glucagon induction of all five genes was comparable to control cells (**Fig 4**). Both constructs were expressed at similar levels (**S3 Fig**), excluding expression differences as a confounding factor.

**Fig 5:**
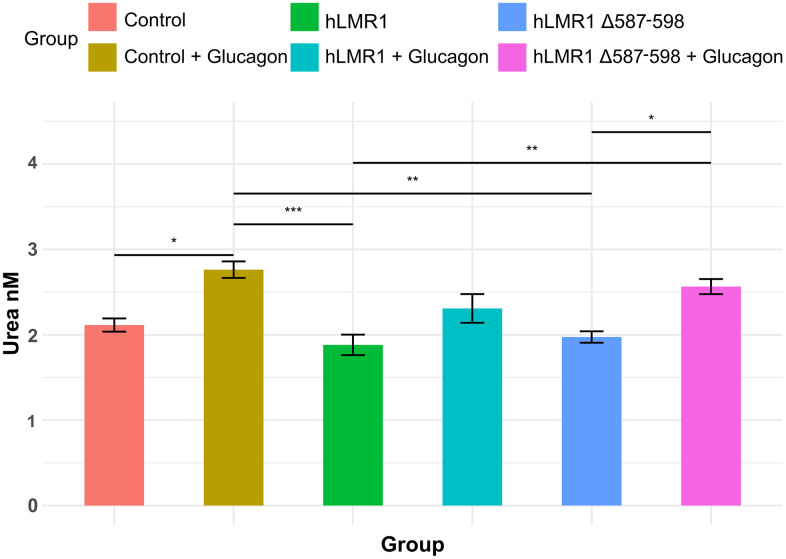
Urea levels in the cell culture medium of cultured primary human hepatocytes as in Fig 4. Data are shown as the geometric mean ± SEM (n = 3-4 for each group). One-way ANOVA statistical analysis and Tukey HSD test were performed to calculate p-values. *, p < 0.05.

Consistent with the transcriptional data, glucagon significantly increased urea production in control and hLMR1Δ587–598-expressing hepatocytes, but not in those overexpressing hLMR1 (**Fig 5**). These results indicate that hLMR1 suppresses glucagon-stimulated amino acid catabolism and ureagenesis likely through direct interaction with target pre-mRNAs.

**Fig 6:**
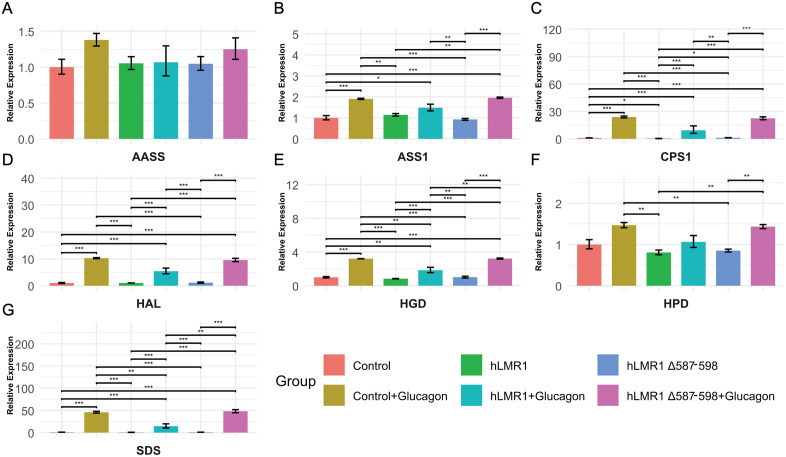
A-G. Relative mRNA expression of AASS, ASS1, CPS1, HAL, HGD, HPD and SDS in primary human hepatocytes (donor 2) infected with empty adenoviruses (Control) or adenoviruses expressing hLMR1, or hLMR1Δ587-598. 36 hours after adenovirus infection, cells were treated with or without glucagon 50nM for 10 hours and RNA were extracted for gene expression analysis. Data are shown as the geometric mean ± SEM (n = 3-4 for each group). One-way ANOVA statistical analysis and Tukey HSD test were performed to calculate p-values. *, p < 0.05, **, p < 0.01, ***, p < 0.001.

To exclude the possibility that our observation was limited to a specific donor, we performed the experiment in human primary hepatocytes from a second donor. As shown in **Fig 6**, we observed a similar result: glucagon robustly induced AA catabolism genes in control hepatocytes and hepatocytes expressing hLMR1Δ587–598. In contrast, glucagon-induced expression of SDS and HGD was significantly suppressed (**Figs 6E & 6G**), and a trend of suppression for ASS1, CPS1, and HAL was observed in hepatocytes overexpressing hLMR1 (**Figs 6B**, **6C & 6D**). We also found the overexpression of hLMR1 and hLMR1Δ587–598 was comparable in this setting (**S4 Fig**). These results support that the role of hLMR1 in suppressing AA catabolism is not limited to a specific donor or genetic background.

### hLMR1 does not repress amino acid catabolism in mouse hepatocytes

**Fig 7:**
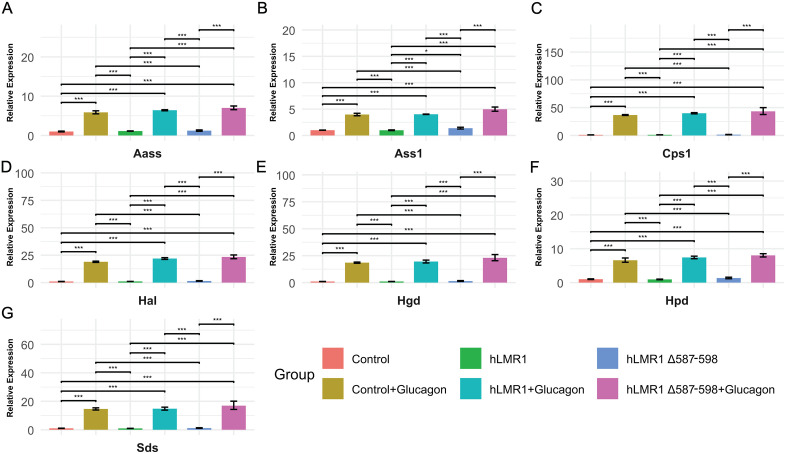
A-G. Relative mRNA expression of Aass, Ass1, Cps1, Hal, Hgd, Hpd and Sds in primary mouse hepatocytes infected with empty adenoviruses (Control) or adenoviruses expressing hLMR1, or hLMR1Δ587-598. 36 hours after adenovirus infection, cells were treated with or without glucagon 10nM for 10 hours and RNA were extracted for gene expression analysis. Data are shown as the geometric mean ± SEM (n = 3 for each group). One-way ANOVA statistical analysis and Tukey HSD test were performed to calculate p-values. *, p < 0.05, **, p < 0.01, ***, p < 0.001.

Given that hLMR1 is a human-specific lncRNA, we next examined whether its overexpression could similarly suppress amino acid catabolism in mouse hepatocytes. Primary mouse hepatocytes were infected with adenoviruses expressing hLMR1 or hLMR1Δ587–598 and treated with glucagon as above. Despite efficient transgene expression (**S5 Fig**), neither construct altered glucagon-induced activation of amino acid catabolism or ureagenesis genes (**Fig 7**). These findings confirm that the repressive function of hLMR1 is species-specific.

### Hepatic hLMR1 expression inversely correlates with amino acid catabolism genes in MASLD

**Fig 8:**
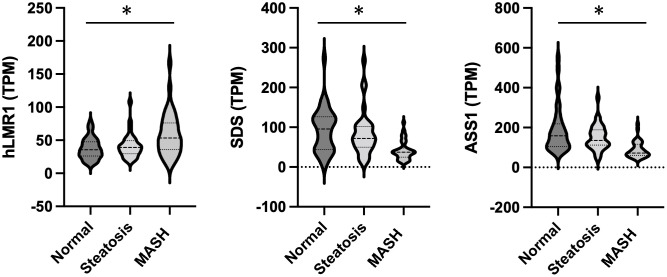
Relative RNA expression of hLMR1, SDS and ASS1 in human liver tissue samples from health individuals (Normal), individuals with hepatic steatosis, and MASH. TPM, transcript per million. * p < 0.05, by one-way ANOVA analysis. Data were analyzed from raw data of NCBI BioProject: PRJNA512027.

To evaluate the physiological relevance of hLMR1, we analyzed a large RNA-seq dataset from liver biopsies across the MASLD spectrum [37]. We found that hLMR1 expression was increased from healthy controls to metabolic dysfunction-associated steatohepatitis (MASH), an advanced stage of MASLD (**Fig 8**). In contrast, representative amino acid catabolism and ureagenesis genes, including SDS and ASS1, were reciprocally downregulated. In an independent interventional study [38], one week of low-carbohydrate dietary treatment reduced hepatic hLMR1 expression while increasing SDS, GLS2, AASS, ASS1, and CPS1 expression in obese MASLD patients (**Table 4**). These clinical observations support that elevated hLMR1 may contribute to impaired amino acid catabolism and ureagenesis in MASLD.

**Table 4:** Hepatic expression of hLMR1 and representative amino acid catabolism genes in obese individuals with NAFLD before and after a low carb diet intervention. Data were reanalyzed from NCBI dataset GSE107650 and a paired t test was performed to calculate the p value.

Gene	Log2(fold_change) After vs Before	P-Value
hLMR1	−0.733363659	7.9371E^-5^
SDS	0.692498252	0.01168179
GLS2	1.127643149	3.1486E^-16^
AASS	0.730033593	0.00243507
ASS1	1.045257926	3.3781E^-12^
CPS1	1.421943193	2.2768E^-6^

## Discussion

Maintaining systemic nitrogen homeostasis is a fundamental function of the liver. Hepatocytes catabolize major non-branched amino acids, including alanine, glutamine, and serine, and couple this process with ureagenesis to safely eliminate ammonia from circulation. However, hepatic capacity for amino acid catabolism and urea synthesis is markedly reduced in metabolic diseases such as Metabolic Dysfunction-Associated Steatotic Liver Disease (MASLD) and type 2 diabetes (T2D) [2–7,9,39]. Recent studies have highlighted disruption of the liver–α-cell axis as a shared feature of both disorders, yet the molecular mechanisms underlying the loss of glucagon-driven amino acid catabolism remain unclear. Here, we identify the human hepatocyte-specific long noncoding RNA (lncRNA), hLMR1, as a key regulator of this pathway. Our findings, together with clinical data, suggest that upregulation of hLMR1 contributes to impaired amino acid catabolism and ureagenesis in MASLD.

Multiple lines of evidence support this conclusion. First, hLMR1 is highly and specifically expressed in hepatocytes (**Fig 1**), consistent with the hepatocyte-specific nature of amino acid catabolism and ureagenesis. Second, under physiological conditions, hLMR1 expression is suppressed by fasting, whereas amino acid catabolism genes are strongly induced, suggesting reciprocal regulation [14,15]. Third, hLMR1 is predominantly localized in the nucleus of human hepatocytes [15], consistent with its proposed mechanism of interacting with the pre-mRNAs of amino acid catabolism genes to repress their transcription. Fourth, in human MASLD patients subjected to a low-carbohydrate dietary intervention [38], hepatic hLMR1 expression was reduced, while amino acid catabolism genes were upregulated (**Table 4**)—aligning with our prior observation that glucose availability promotes hLMR1 expression [40].

Our mechanistic data support a model in which hLMR1 directly interacts with the pre-mRNAs of amino acid catabolism genes to suppress their expression. Among the most strongly regulated targets is SDS, whose first intron contains several hLMR1-interacting motifs. Similar lncRNA–pre-mRNA regulatory mechanisms have been described previously, where lncRNAs act as organizing hubs to coordinate the expression of functionally related genes [41]. Our results extend this concept to hepatic nitrogen metabolism: amino acid catabolism and ureagenesis genes are highly expressed during fasting, when glucagon-driven transcription predominates, and must be efficiently silenced upon feeding. We propose that feeding-induced hLMR1 acts as a nuclear scaffold that binds pre-mRNAs of these genes to repress their expression, thereby enabling rapid metabolic switching between fasting and fed states.

Consistent with its species specificity, hLMR1 failed to suppress glucagon-induced amino acid catabolism in primary mouse hepatocytes, indicating that its regulatory function is unique to humans (**Fig 7**). Whether a murine homolog exists or alternative mechanisms fulfill an equivalent role in mice remains unknown, suggesting an additional regulatory layer for nitrogen metabolism that evolved specifically in humans.

Several important questions remain. As hLMR1 regulates both lipid and amino acid metabolic pathways, future studies should define the precise RNA–RNA and RNA–protein interactions underlying its function. Mapping hLMR1’s structural domains and testing how specific mutations affect target recognition will be essential to determine whether secondary RNA folding contributes to its regulatory activity. Another key direction is to assess whether inhibition of hLMR1 can restore the impaired liver–α-cell axis observed in MASLD and T2D [4,9,39]. Given that hLMR1 promotes hepatic cholesterol synthesis and is expressed exclusively in hepatocytes, pharmacological suppression of hLMR1—using antisense oligonucleotides or GalNAc-conjugated siRNAs—could simultaneously ameliorate hyperlipidemia and hyperglycemia. Because hLMR1 is human-specific, such therapeutic strategies can only be evaluated in diet-induced MASLD/T2D models in humanized liver mice [42–44] before clinical studies.

The limitations of our study include: 1. RNA-seq analysis using humanized liver tissues. First, RNA-seq was performed on total RNA from humanized livers containing both transplanted human hepatocytes and residual mouse cells. We therefore discard reads that cannot be uniquely assigned to the human or mouse genome; reads from highly conserved genes may be lost, reducing power for differential expression. Second, the humanized liver model has greater biological variability than pure-species models because human hepatocyte repopulation in TK-NOG mice varies (~15–80% by circulating human albumin). This heterogeneity increases variability in gene expression measurements. 2. As human samples are inherently diverse, additional experimental validation using hepatocytes from multiple donors would further strengthen our conclusions. 3. Other limitations. (a) the use of pooled liver tissue for ChIRP, which decreases inter-individual variability but may obscure donor-specific effects; (b) reliance on overexpression and knockdown approaches that may not faithfully reproduce endogenous hLMR1 expression levels or regulatory context; and (c) absence of comprehensive in vivo loss-of-function validation in humanized models beyond the initial knockdown screen.

## Supporting information

S1 Fig PCA of VST-normalized counts showing PC1 (46% variance) and PC2 (18% variance). Samples cluster by group: sh_lacZ (red) and sh_hLMR1 (teal), with sample labels indicated. (TIFF)

S2 Fig A. Regular PCR validating the human-specific real-time PCR primers. H: human hepatocytes; M: mouse hepatocytes. B. Real-time PCR gene expression in humanized mice receiving adenovirus for control (sh-lacZ, n = 4) or knockdown of hLMR1 (sh-hLMR1, n = 5). A human-specific RPL13A primer was used for normalization. p values were determined by multiple student t test and marked in the Figure.
(TIFF)

S3 Fig Relative expression of hLMR1 among groups as in Fig 4. (TIFF)

S4 Fig Relative expression of hLMR1 among groups as in Fig 6. (TIFF)

S5 Fig Relative expression of hLMR1 among groups as in Fig 7. ND: Non-detected.
(TIFF)

S1 Table List of genes that were *differentially regulated* by knocking down of hLMR1 in humanized livers. (XLSX)

S2 Table List of top 1000 peaks and their associated genes in hLMR1 ChIRP RNA-seq analysis. (XLS)

S3 Table Sequences of ChIRP probes, cloning primers, and real-time PCR primers used in this study. (XLSX)
