# Supplementary figures and images for: hLMR1, a hepatocyte-specific long noncoding RNA that represses amino acid catabolism through pre-mRNA interaction in human liver
Source: PLoS One. 2026 Jul 21;21(7):e0353674. doi: 10.1371/journal.pone.0353674 (PMC13387515; doi:10.1371/journal.pone.0353674)

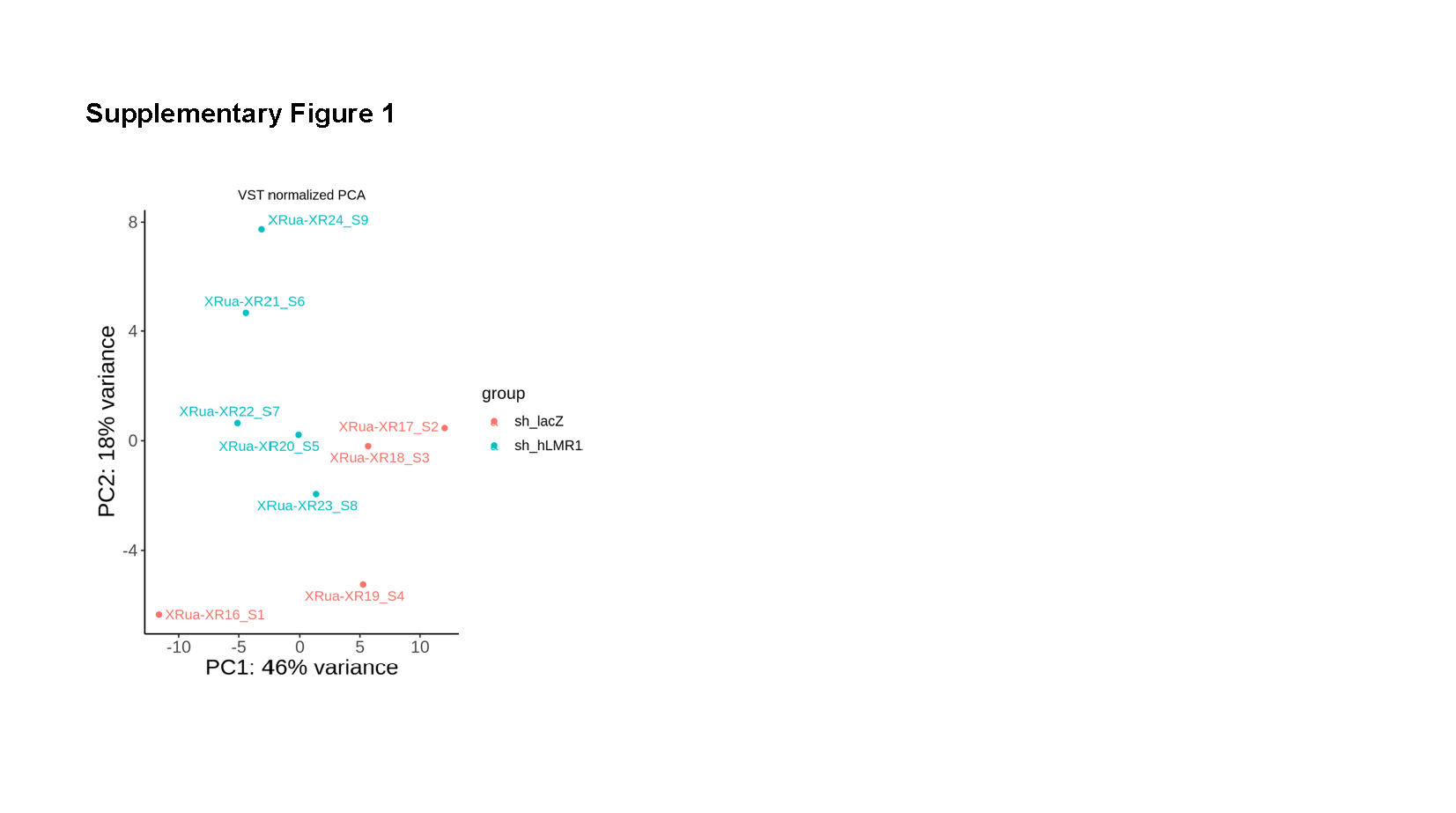

Supplement: S1 Fig — (TIFF) [file pone.0353674.s001.tiff]

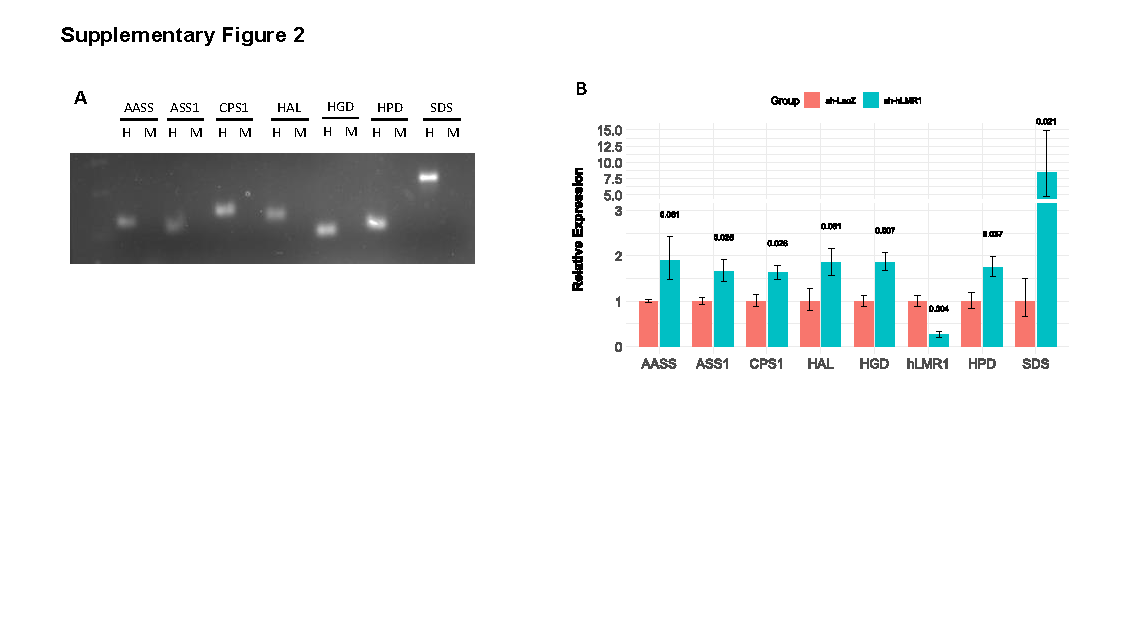

Supplement: S2 Fig — H: human hepatocytes; M: mouse hepatocytes. B. Real-time PCR gene expression in humanized mice receiving adenovirus for control (sh-lacZ, n = 4) or knockdown of hLMR1 (sh-hLMR1, n = 5). A human-specific RPL13A primer was used for normalization. p values were determined by multiple student t test and marked in the Figure. (TIFF) [file pone.0353674.s002.tiff]

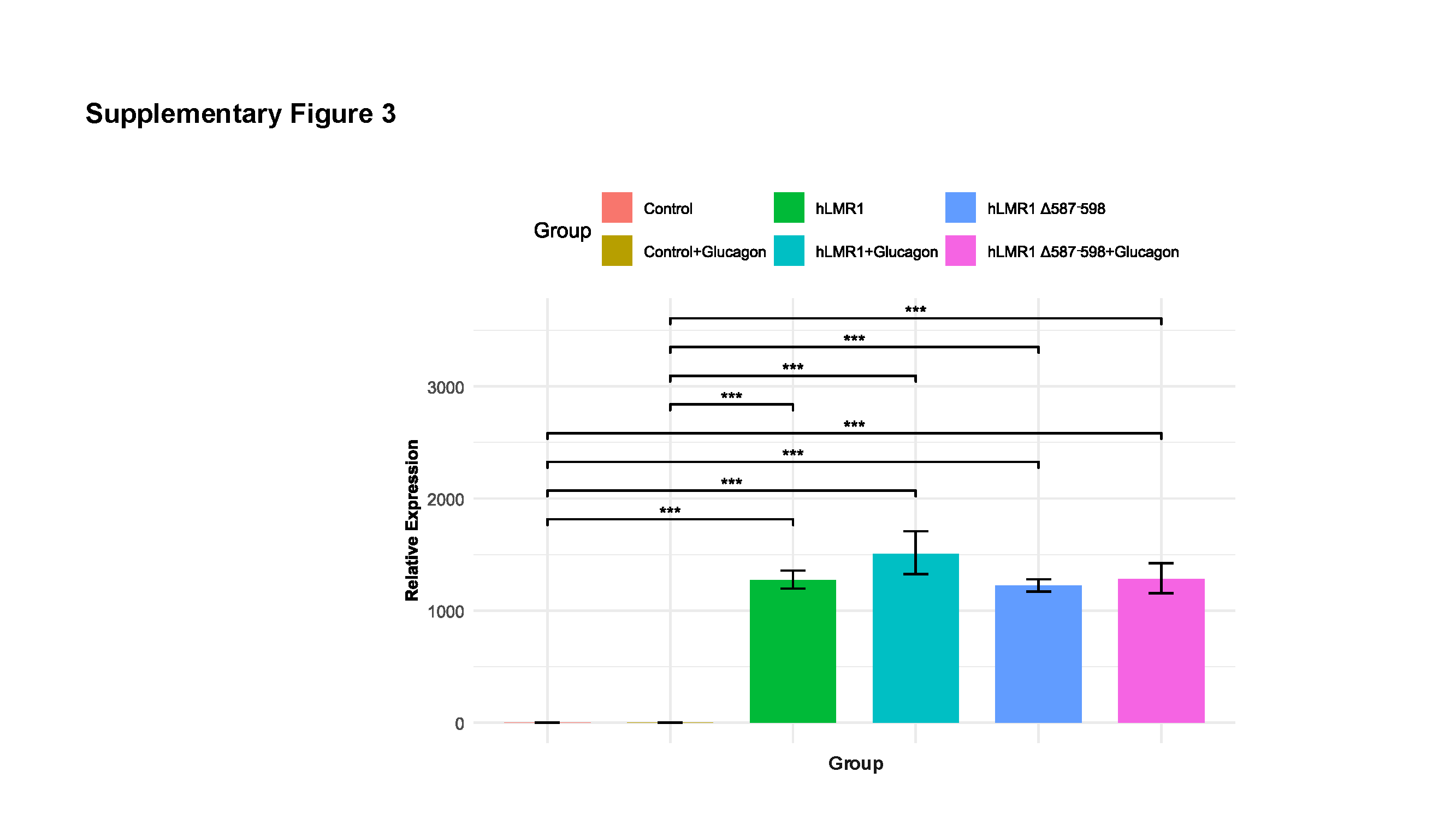

Supplement: S3 Fig — (TIFF) [file pone.0353674.s003.tiff]

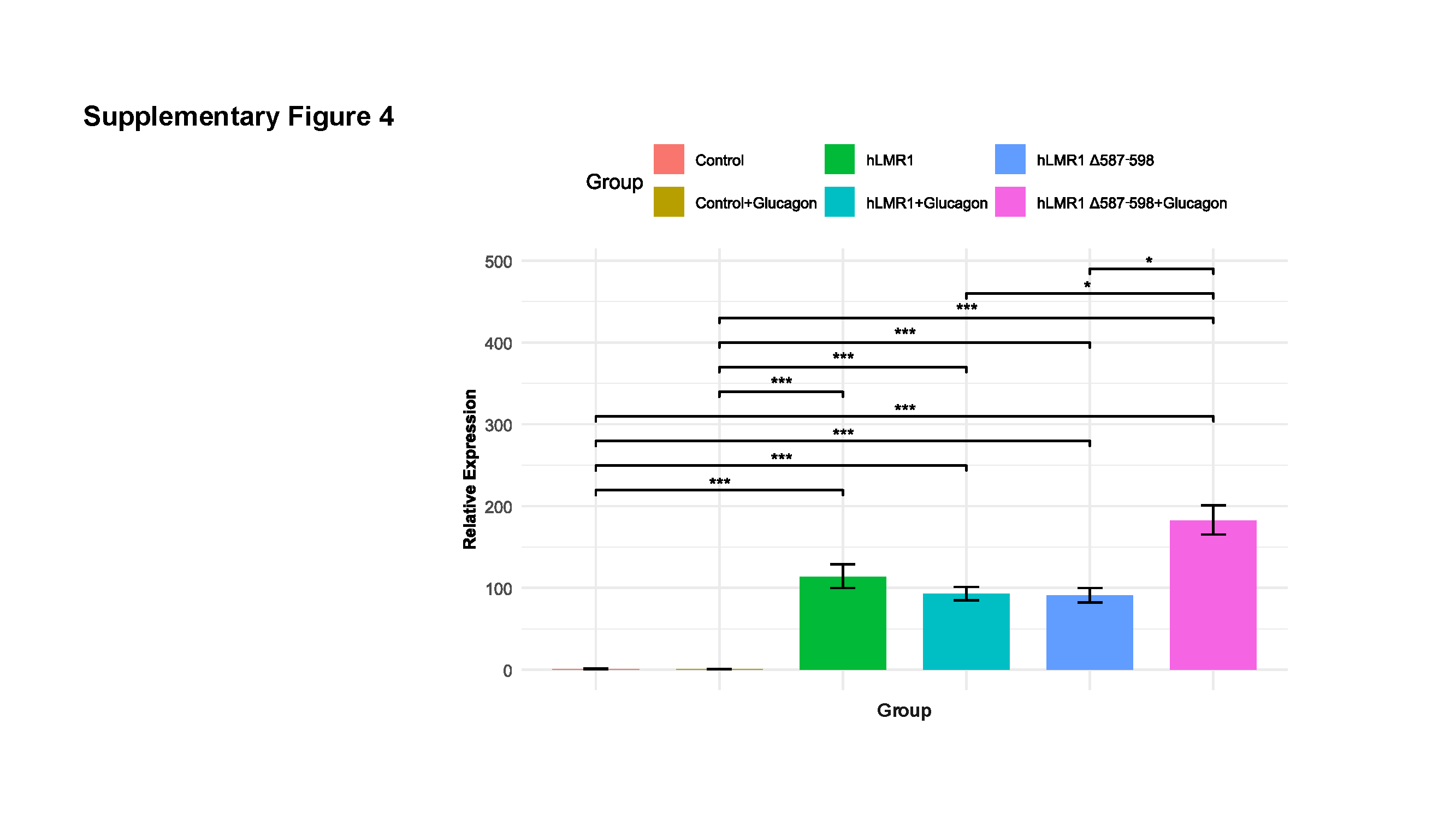

Supplement: S4 Fig — (TIFF) [file pone.0353674.s004.tiff]

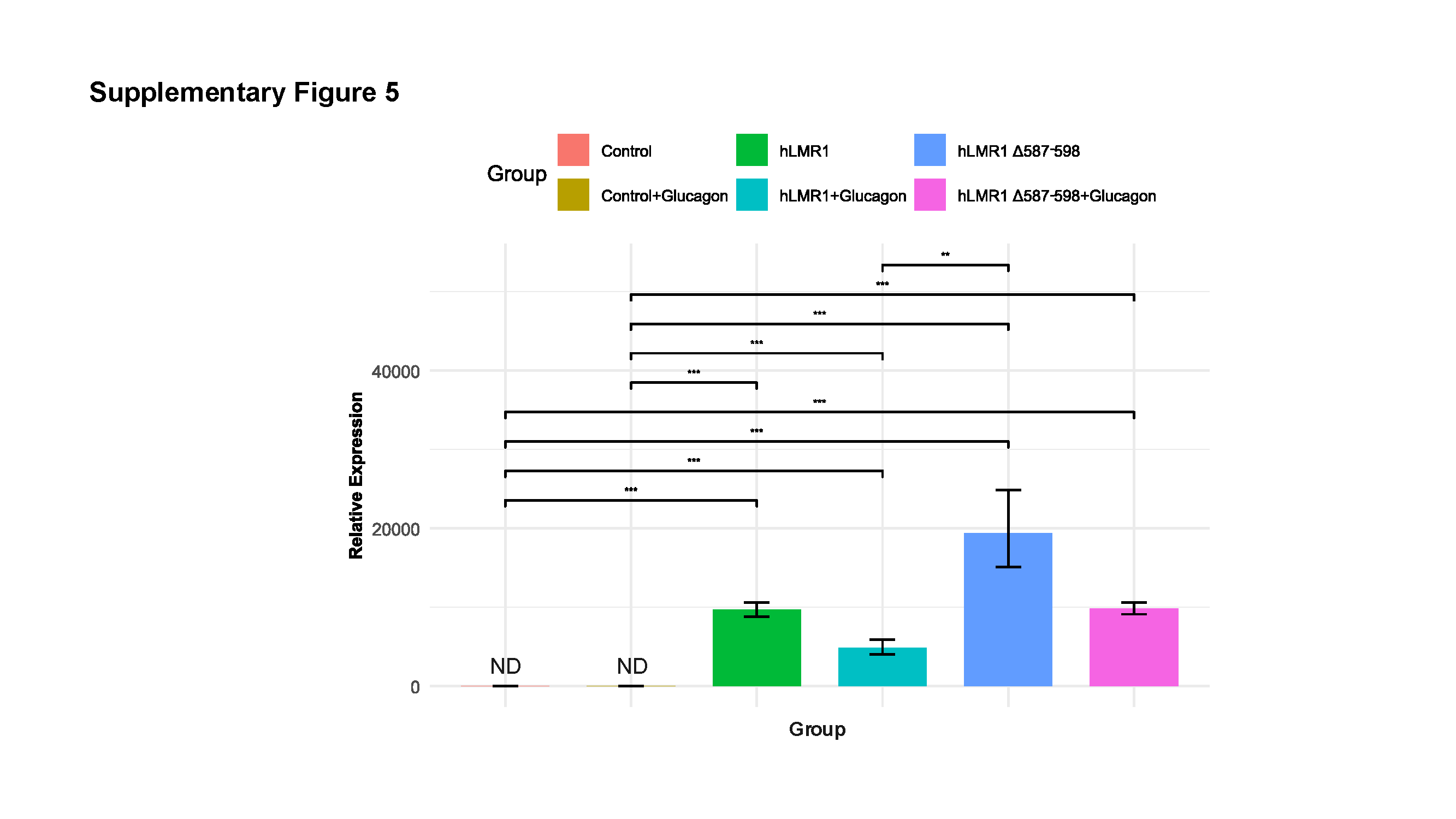

Supplement: S5 Fig — ND: Non-detected. (TIFF) [file pone.0353674.s005.tiff]
